# Health system readiness to support facilities for care of preterm, low birth weight, and sick newborns in Ethiopia: a qualitative assessment

**DOI:** 10.1186/s12913-019-4672-2

**Published:** 2019-11-21

**Authors:** Abubeker Kedir Usman, Eskinder Wolka, Yared Tadesse, Abraham Tariku, Abate Yeshidinber, Alula M. Teklu, Kirsten Senturia, Wendemaghen Gezahegn, James A. Litch, And the Every Preemie–SCALE Ethiopia Implementation Research Collaboration Group, Hagos Gidey, Tedros Hailu, Solomie Jebessa, Amaha Kahsay, Kemal A. Kuti, Gillian A. Levine, Judith Robb-McCord, Alemu Tesfahun, Berhe Dessalegn Tuamay

**Affiliations:** 1 Madda Walabu University, Bale Robe, Ethiopia; 2 Wolaita Sodo University, Wolaita Sodo, Ethiopia; 3 Federal Ministry of Health, Addis Ababa, Ethiopia; 4 St. Paul’s Hospital Millennium Medical College, Addis Ababa, Ethiopia; 5 Global Alliance to Prevent Prematurity and Stillbirth (GAPPS), 19009 33rd Avenue W, Suite 200, Lynnwood, WA 98036 USA

**Keywords:** Health system, Health system readiness, Preterm, Low birth weight, Sick newborn, Newborn, Newborn health, Qualitative research, Ethiopia

## Abstract

**Background:**

Preterm birth is a worldwide challenge with the highest burden in low- and middle-income countries. Despite availability of low-cost interventions to decrease mortality of preterm, low birth weight, and sick newborns, these interventions are not well integrated in the health systems of low- and middle-income countries. The aim of this study was to assess, from the perspective of key stakeholders comprising leaders in the public health system, the health system readiness to support health care facilities in the care provided to preterm, low birth weight, and sick newborns in different regions of Ethiopia**.**

**Methods:**

A qualitative assessment using in-depth interviews with health facility leaders was conducted in health facilities in 3 regions of Ethiopia from December 2017 to February 2018. The interview guide was developed using a modified version of the World Health Organization health system building blocks.

**Results:**

Across the public health system, adequate and reliable space, power, and water were problematic. Human resource issues (training, staffing, and retention) were critical to being able to properly care for preterm, low birth weight, and sick newborns. Problems with functional equipment and equipment distribution systems were widespread. Funds were lacking to support preterm, low birth weight, and sick newborn needs in facilities. Data collection practices, data quality, and data utilization were all problematic. There were gaps in the availability of guidelines and protocols, specifically targeting preterm, low birth weight, and sick newborn care. Key facilitators, information disseminators, and influencers identified in the study were the Health Development Army, community and religious leaders, and mothers and families who had had positive experiences or outcomes of care.

**Conclusions:**

The Ethiopian health system has opportunities across all 7 World Health Organization health system building blocks to strengthen readiness to support health facilities to provide quality care and improve outcomes for preterm, low birth weight, and sick newborns.

## Background

It is estimated that 15 million (11.1%) babies born worldwide in 2010 were preterm (born alive before 37 weeks gestation) [1]. Preterm birth is a worldwide challenge, with the highest burden in low- and middle-income countries (LMICs). Despite the availability of low-cost interventions to reduce mortality of preterm, low birth weight (LBW), and sick newborns, these interventions are not well integrated in the health systems of LMICs. Neonatal deaths are the leading cause of under-5 mortality [2], and complications of prematurity is the leading cause of neonatal mortality [3, 4].

Through substantial investments in public-sector health systems, Ethiopia has achieved notable progress over the last decade in preventing postnatal newborn deaths. Newborns benefit from high-impact interventions that include integrated community case management, community-based newborn care, and prevention of mother-to-child transmission of HIV through Option B+. However, reductions in neonatal mortality have lagged behind reductions in child mortality [4]. The country has one of the highest neonatal mortality rates globally, with 28 neonatal deaths per 1000 live births, and has the sixth highest number of neonatal deaths, with an estimated 90,000 deaths in 2016 [5]. Of under-5 deaths in Ethiopia, 44% occur within the first 28 days of life, a rate that has remained stagnant. The most common causes of death among newborns in Ethiopia are prematurity (37%), infection (28%), and asphyxia or other intrapartum events (24%) [6].

Countries with high neonatal mortality have bottlenecks in almost all of the health system building blocks defined by the World Health Organization (WHO): service delivery, health workforce, health management information systems, access to essential medicines, financing, leadership and governance, and community engagement [7, 8]. A study from 12 high-burden countries showed that the most prevalent bottlenecks were in the areas of health workforce and health financing (10 out of 12 countries), followed by community ownership and partnership (9 out of 12 countries) [9].

Establishment of neonatal intensive care units (NICUs) and diversified levels of care have increased the survival of preterm, LBW, and sick newborns less than 32 weeks of gestation, although this may not apply to most countries with the highest burden of preterm deliveries and deaths [10]. Despite the absence of these high-level centers, moderately preterm newborns can be saved with feasible, cost-effective care [11]. Barriers to neonatal care include shortages of qualified personnel, geographical inaccessibility, inadequate regulation of pharmaceutical and private sector providers, insufficient provider knowledge and skills, poor demand for interventions, and lack of equipment and infrastructure. Continuous and multifaceted improvement in the established health system improves the overall outcome of neonates [12, 13].

The aim of this study was to assess, from the perspective of key stakeholders comprising leaders in the public health system, the Government of Ethiopia health system’s readiness to support health care facilities in the care provided to preterm, LBW, and sick newborns in 3 different regions of Ethiopia. For the purposes of this paper, we define *health system readiness* as the preparedness of the system, including *all* components—facilities, staff, and organizational structure—to provide appropriate evidence based care for preterm, LBW, and sick newborns primarily from 28 weeks gestational age and above.

## Methods

### Study design

A team of researchers from Ethiopia and the United States conducted a qualitative assessment in multiple sites across Ethiopia to describe facility readiness to deliver care services to preterm, LBW, and sick newborns, by assessing service availability and key processes.

From December 2017 to February 2018, data collectors conducted key informant interviews with facility leaders in the public health care system, many of whom were also clinical care providers delivering obstetric and newborn care services in study health facilities. Qualitative methods were used to gather rich data to inform *how* and *why* the health system needs improvements in serving preterm, LBW, and sick newborns, rather than to establish prevalence numbers. Taking a narrative approach, we used thematic content analysis to answer specific a priori questions about the Ethiopian health system. This report conforms to the Standards for Reporting Qualitative Research [14].

The WHO health systems building blocks provide a framework for understanding and assessing the key components of a health system. Within each block, key domains and the critical factors that influence structure, function, and outcome of that domain are described. This study used a modified version of the health systems building block framework to guide our assessment of the current context of services for preterm, LBW, and sick newborns in Ethiopia [15].

### Study sites

The Federal Democratic Republic of Ethiopia is among the most populated countries in Africa, with a population estimate of 107 million for 2018 [16]. One urban and 2 rural regions were selected to allow for assessment and description of different settings and contexts in Ethiopia representing an urban community, a settled agrarian community, and a semi-pastoral agrarian community.

Staff and clients from a subset of health facilities at each tier of the public health care system in each region participated, including specialized hospitals, general hospitals, primary hospitals, health centers, and health posts [17].

### Data tools

A new tool was developed for this project using a multi-step process. First, based on the WHO framework, the scope of the tool was defined, and questions were drafted. After their initial training, data collectors pre-tested the instrument at St. Paul’s Hospital Millennium Medical College with 5 interviewees similar to the target sample. Adjustments were made for flow, content, terms used, prompts, and instructions.

Using this structured interview guide, facility leaders were asked questions about policies and guidelines, programs, facility preparedness, and referral transfer systems, all with respect to preterm, LBW, and sick newborns (see Additional file 1).

### Sampling

We used purposive convenience sampling to recruit from all clinical, nursing, and administrative leaders who were involved in the oversight of units or care programs for pregnancy, labor, and delivery/obstetrics, and gynecological, postnatal, and newborn care services at the study health facilities. We interviewed 37 leaders from all public specialized, general, and primary hospitals and a subset of health centers and health posts located within the 3 study regions (see Table 2).

### Saturation

Interview data were reviewed from all 3 study regions periodically during data collection until data saturation [18] was reached, according to the judgment of the core team.

### Research team composition, training, and supervision

The research team was composed of core members, data collectors, and data analysts. Experienced data collectors were trained on the study overview, objectives, participant selection, instruments, and interview skills. Supervision was conducted throughout data collection in all 3 study regions using a supervision checklist.

### Data collection and management

#### Recruitment

Trained study interviewers identified facility administrators who identified department leaders eligible to participate in the study. Interviewers invited and scheduled potential participants for in-person interviews.

#### Location

Each interview was conducted in the leader’s office at a time convenient for the interviewee. Participants were offered their choice of languages (Amharic or Oromiffa) for the interview.

#### Recording, transcribing, and translation

Demographic data were recorded on a tablet and interviews were recorded digitally. Recordings were transcribed into the local written language by experienced transcriptionists and were subsequently translated by professional translators. Transcriptions and translations were spot-checked for accuracy by a third team member. Every effort was made to maintain participants’ confidentiality both during data collection and during report writing and publication. All audiotapes were destroyed immediately following transcription. No names are attached to any of the data. In the results, quotes are identified only by location (AMH = Amhara, ORO=Oromia, AA = Addis Ababa) where relevant, and by facility level (HP = health post, HC = health center, HOSP = hospital) where relevant to the findings.

### Analysis

Data were entered into NVivo version 12 and analyzed using thematic content analysis [19]. Researchers involved in the study design and qualitative coders developed a codebook and applied it to the coding of all transcripts.

All transcripts were open-coded using the final version of the codebook to capture key themes and relevant ideas as identified in the data. Each transcript was coded by 2 separate coders, and disagreements were resolved by the lead analyst who reviewed all discrepancies and discussed them with the second coder as necessary to reconcile coding. Once coding was complete, code reports were produced for each code, cleaned, and prepared for synthesis.

Each code report was synthesized by one team member as follows: initially, text excerpts no longer appearing relevant to the code were grayed out; remaining excerpts were annotated with comments; particularly illustrative quotes were highlighted; and comments were summarized into a table of theme domains and subdomains with associated quotes for each code report.

## Results

### Description of sample

All respondents were from Addis Ababa, Amhara Region, and Oromia Region. The characteristics of study regions are shown in Table 1.

**Table 1:** Characteristics of study regions^a^

Characteristics	National	Addis Ababa	Amhara	Oromia
Demographic indicators				
Population, No.^b^	73,918,505	2,738,248	17,214,056	27,158,471
Proportion urban population, %^b^	16.2	100.0	12.3	12.4
Total fertility rate, No. of children per woman	4.6	1.8	3.7	5.4
Proportion of women who are literate, %	42.0	87.8	44.9	37.3
Proportion of women who own/use a bank account, %	15.1	53.6	20.9	8.4
Proportion of women who own/use a mobile phone, %	27.3	87.0	21.2	23.3
Proportion of men who are engaged in agriculture, %	71	1	62	41
Mortality rates				
Under-5 mortality, No. per 1000 live births	67	39	85	79
Infant mortality, No. per 1000 live births	48	28	67	60
Neonatal mortality, No. per 1000 live births	29	18	47	37
Low birth weight rate, %	12.7	11.5	22.2	13.1
Maternal and child health services indicators				
Proportion of pregnant women who received antenatal care from a skilled provider, %	28.0	96.8	67.1	50.7
Proportion of deliveries in a health facility, %	26.0	96.6	27.1	18.8
Proportion of women with a postnatal checkup in first 2 d after birth, %	17.0	55.4	21.9	11.8
Proportion of children (ages 12–23 mo) who received all basic vaccinations by 12 mo, %	22.0	81.6	39.9	24.3

^a^Data from the Ethiopia Demographic and Health Survey 2016, except as denoted in footnote b [20]

^b^Data from the 2007 National Census [21]

Clinical leaders were proportionally divided across all 3 regions; 50% were 26 to 30 years of age; and all were employed as midwives, nurses, health officers, heath extension workers (HEWs), or neonatologists (Table 2).

**Table 2:** Background characteristics of facility leaders

Characteristics	Leaders, No. (*n* = 37)	Percentage
Age, y^a^		
20 to 25	6	16.7
26 to 30	18	50.0
31 to 35	6	16.7
36 to 40	4	11.1
41 to 45	2	5.6
Sex		
Female	21	56.8
Male	16	43.2
Profession		
Midwife	1	2.7
Nurse	15	40.5
Health officer	7	18.9
Health extension worker	13	35.1
Neonatologist	1	2.7
Position		
Head nurse	1	2.7
Head, health center	5	13.5
Head, health post	15	40.5
Head, maternal child health	1	2.7
Head, NICU	3	8.1
Head, pediatric department	1	2.7
Lead, labor and delivery	1	2.7
Medical director	9	24.3
Coordinator, NICU	1	2.7
Facility level		
Specialized hospital	2	5.4
General hospital	3	8.1
Primary hospital	1	2.7
Health center	16	43.2
Health post	15	40.5
Region		
Addis Ababa	12	32.4
Amhara	14	37.8
Oromia	11	29.7

^a^One participant not reported

*Abbreviation: NICU* neonatal intensive care unit

### Health service delivery

#### Infrastructure

Across the public health system, adequate and reliable space, power, and water functionality were problematic. Table 3 displays the issues linked by facility levels.

**Table 3:** Space, power, and water functionality across facility levels

Facility Level	Problem
Space	
Health post	Only space for immediate assessment and referral
	1-room facilities
	No separate space for mothers, neonates, or preterm, LBW, and sick newborns
	Condition of building compromised (roof caved in)
	Lack of fully equipped rooms
Health center	Size of existing rooms inadequate
	Could not serve multiple preterm, LBW, and sick newborns who arrived simultaneously
	No inpatient or pediatric unit
	Could not accommodate newborn when mother was getting care and unable to care for her newborn
	Necessary to refer cases beyond first aid due to lack of space
	Multiple activities usually combined in 1 or 2 rooms
	L&D shared with postnatal ward; space limited and uncomfortable
	No postnatal room; newborns with mothers in waiting room
	Preterm newborns cared for in delivery room
	No space for parent to rest or sleep when newborn was admitted for care
	Antenatal care, family planning, Integrated Child Illness in 1 room; prenatal and pre-labor in another room
	No separate room for neonates or preterm, LBW, and sick newborns
	Shortage of rooms for delivery
Primary hospital and general hospital	Shortage of space in NICU; had to serve only most critically sick newborns
	No space for newborns in delivery rooms
	The only space for breastfeeding mothers was in a separate building away from the NICU
	Space for doctors and nurses was crowded
	KMC room had no sink and limited beds
	Delivery room lacked beds; mothers on floor mattresses
	Shortage of beds in mini-NICU; preterm, LBW, and sick newborns shared beds “Laying 4 and 5 kids on 1 bed is very difficult. That is how we are using it. I think that’s why our work is not effective. Preterm infants that come [to] us rarely survive because both the septic and the healthy sleep together” (ORO-HOSP).
	KMC babies and mothers in the delivery room with others
	Neonatal room did not meet standards
Specialized hospital	No space for parents in NICU; they had to sleep outdoors or in the latrine
	Sometimes had to refer preterm, LBW, and sick newborns due to shortage of beds
	Multiple babies in 1 incubator
	Neonates put into adult and surgical wards due to lack of space
Power	
Health post Health center Primary hospital	Power interrupted and unreliable “There [are times that] the power will be off up to 2 days. Even there was time that we take our patients to other hospital by ambulance due to lack of power” (AA-HOSP)
	Generator and/or solar power do not ensure uninterrupted supply Power/generator failure can also result in water failure
Water	
Health post	Only periodic water
Health post Health center	Reliance on river water
	No water of any kind whatsoever
	Only 3 reported that water was continuous/uninterrupted, with 1 due to mountain location
	Collected and used rain water
Health center General hospital and specialized hospital	Collected and used unclean river water “We don’t have clean tap water. We have to get it from the river. Mind you the kinds of infections and other waterborne diseases that may be caused as a result of this” (ORO-HC)
	Collected and used water from a nearby source; not available on-site
	Even in a new health center facility, water pipes had leaks; others reported broken pipes
	Water came only on alternate days
	Mothers not able to clean up after birth “It is difficult for a mother to go home covered in blood after birth. .. For example, if a mother gives birth here on dusty space, then it is no different from giving birth at home” (AMH-HC)
	Shortages for 1 to 2 weeks “We may not get water for 1 or 2 weeks. To eat our food, we have to buy packed water. Even we do not get to wash our hands. We prepared large water container, so we fetch from that. .. We have to carry from the ground [floor] to the second floor” (AA-HOSP)
	Periodic interruptions due to an aged building
	Parents restricted from visiting preterm, LBW, and sick newborns in NICU if there was no water to wash visitor gowns

#### Service readiness

Participants were asked how ready their local health system was to deliver (1) routine care to newborns and (2) care to preterm, LBW, and sick newborns—across labor and delivery, NICU, kangaroo mother care (KMC), and inpatient wards. Only hospitals and advanced health centers have specialized care units, but all leaders were asked about readiness for newborn care and preterm, LBW, and sick newborn care. A few leaders reported being very equipped to provide care, but most cited problems in one or more areas that inhibited effective provision of care. Table 4 shows the challenges related to readiness in each of the facility units.

**Table 4:** Readiness to provide newborn care and preterm, LBW, and sick newborn care

Facility Unit	Facility Level	Readiness Challenge
Labor and delivery	All	Variable across facility levels and between facility
	Health post	Delivery room not expected nor equipped to provide neonatal service
		Provided care for emergency patients only; non-emergency patients referred to health center
		Lacked qualified providers for newborn care
	Health center	Provided care in L&D but only had 1 room and needed more space
		Provided the service at the time needed but space and comfort were inadequate
		Equipped for neonatal care and for preterm, LBW, and sick newborns, but health center rarely encountered them
		Served routine-care neonates but not preterm, LBW, and sick newborns; no materials or specialized provider
		Had laboratory technician and new rooms but lacked additional professionals
		Prepared, but preterm, LBW, and sick newborns transferred to under-5 department or hospital as needed
		Space limitations prohibited neonatal care in L&D
		Inadequate supplies, services, and space for preterm, LBW, and sick newborn care
	Hospital	Not enough training for L&D providers to give proper neonatal care
		May not have had space for neonates or proper equipment to give care
		Hospital staff was ready but lacked ventilator
		Hospital lacked space and beds; could not accommodate newborns
		Lack of infrastructure, equipment, and food for patients hindered abilities
		L&D rooms lacked handwashing stations
NICU	All	Most respondents reported no NICU; no expectation for that to change
	Health post	Never expected
	Health center	No NICU
	Hospital	Needed supplies and additional providers
		Needed space for parents; no place for them to wait or sleep
		Material shortage, including of medication and equipment
		NICU isolated; parents could look through glass; mother could visit if baby improved; when no water to clean gowns, parents could not enter NICU
KMC	All	Adequate in many facilities; most offered KMC even if referring up
	Health post	KMC service provided prior to referral to health center
		Space may be just a corner or nook or no space at all
	Health center	No separate room; KMC in L&D or pediatrics; little space with no food storage
	Hospital	KMC and NICU joined in this hospital; sophisticated care available, including water and toilet
		KMC room available for dyads when mother was healthy; when mother admitted, baby stayed in KMC without parent
Inpatient ward	Health post	Never expected
	Health center	None; accommodated elsewhere in facility
	Hospital	Facility with NICU could care for preterm, LBW, and sick newborns; budgets may not have covered basics like diapers, clothes, or even necessary treatment for preterm, LBW, and sick newborns

*Abbreviations: KMC* kangaroo mother care, *L&D* labor and delivery, *LBW* low birth weight, *NICU* neonatal intensive care unit

### Health workforce and human resources

Human resource issues (training, staffing, and retention) were critical to being able to properly care for preterm, LBW, and sick newborns.

#### Provider training and skills

Clinical leaders saw staff training as mandatory for quality service provision and specifically recommended training in newborn care, including refresher training that did not currently exist: “There should be constant providers and the number of providers need to be increased; one provider is needed for one baby if they are critically sick; if possible one nurse should be assigned for one baby. To do so, the number of health providers matters” (AA-HOSP). Training on newborn care basics was not common for staff in general. Staff preservice training in the preterm, LBW, and sick newborn program was inadequate for many, if not most, providers. Labor and delivery training was limited and training applicable to preterm, LBW, and sick newborn care was primarily related to breastfeeding and KMC. Previously, nongovernmental organizations had provided some training in care for preterm, LBW, and sick newborns, but that was no longer happening.

Lack of training was identified as one factor leading to unnecessary referrals. Inadequate numbers of trained providers—especially senior staff, skilled professionals, and NICU staff—were also seen as limitations to system readiness. The difficulty of getting a newborn with a complication to an appropriate specialist led to loss of life. Furthermore, high turnover of trained professionals was an ongoing problem.

#### Communication

Functionality also depended upon effective communication between providers in the same facility and in other facilities. Participants reported that meetings were the primary form of communication. Using catchment area meetings at hospitals to bring health center and hospital staff together to communicate or consult on cases seemed widespread. Meetings were also regularly held within facilities to communicate about specific cases or deliver training updates, in the style of grand rounds seminars. Health centers communicated with health posts via midwives and with HEWs via conference meetings. This may have been limited to certain facilities and not universal. Changes to guidelines, policies, and trainings were conveyed through written communication from clinical leaders.

Participants recognized the importance of communication for effective care of preterm, LBW, and sick newborns and called for improvements both within units at facilities and between levels of facilities: “To improve these challenges, I think we need to communicate—I mean, all of us in our respective departments/units. The doctors with the doctors working there, the interns with the interns working there have to closely communicate. The nurses and midwives also have to communicate among themselves on service delivery and on solving the problems” (ORO-HOSP).

Communication followed a hierarchical path when information or expectations were being transmitted from the top of the system down through the various facility levels, as one participant described: “Orders trickle down from the authorities at the zone level to the ones at the *woreda* (district) level, then to the health directory, then to the health center, then finally to the health posts. These orders are usually transmitted orally but there are cases when we file them as written forms. We will be given instructions, updates on policies and regulation so we can apply them to our model. We are also given guidance on the best route to implement the reforms, then we inform the community on the steps to be taken” (ORO-HP). It was not clear how information, requests, and expectations were conveyed from the bottom up in this system.

#### Health extension workers

HEWs were seen as the key link between mothers in the local community and the entire health system through initial identification during pregnancy and postpartum follow-up. In the health system design, Health Development Army volunteers were supposed to identify pregnant women or recent home births and pass that information to the HEWs. HEWs then performed the critical role of home visits and monitoring mothers and babies in the community. They sometimes managed home delivery issues and follow-up care for preterm, LBW, and sick newborns: “Since the primary responsibility of the health extension worker is within the community in rural [areas], they are accountable to follow newborn and maternal health conditions. Them being there will support us, because they will take the responsibility to ensure pregnant mothers initiate follow-up and come to the health center for childbirth. Then we will follow up, calculate the EDD [estimated date of delivery], and identify those who will come for follow-up visits or not. Further, the health center will decide which newborn case can be treated here and transfer those which need a critical follow-up. That is how we do things” (AMH-HC).

HEWs played a critical role in referring preterm, LBW, and sick newborns to higher levels of care, especially in rural areas and especially following home births. Several participants spoke of the need for leadership to recognize this critical role and prioritize it. In some cases, HEWs accompanied patients to higher-level care. When women self-referred to higher care, it was seen as a failure of the HEW identification and tracking system.

Participants made several recommendations to improve the HEW role and functionality in the care of preterm, LBW, and sick newborns:

- Conduct initial and ongoing training and capacity building; this is necessary to increase skills if HEWs take on expanded, more sophisticated roles;
- Improve referral system feedback to strengthen the relationship between HEWs and higher-level facilities;
- Increase supervision and support for HEWs;
- Increase governmental support for the role of HEWs and commitment to sustained improvements; and
- Demonstrate leadership respect for HEWs so that others within the larger system adopt a respectful attitude toward HEWs.

Participants also noted that the performance of HEWs could be improved only within the limits of the current system’s infrastructure; problems related to the functionality of water, power, and roads would limit even the best trained and supported HEWs.

### Health management information systems and data monitoring and evaluation

Data were being collected at multiple levels throughout the health system, but not consistently within levels—nor even within facilities in some cases. All 3 facility levels—health post, health center, and hospital—reported at least one example of using data for performance monitoring, quality improvement, or health trends. Participants were aware that documentation was important, not only for individual patient tracking but also for health trend tracking, such as observing preterm birth rates, for larger system planning.

#### Information production

Data collection practices were problematic on many levels. Primary documentation seemed to be associated with referral, although this may be due to the focus of the interview instrument on referrals. The data collection system was variable, and even erratic, depending on the individual facility and the level of facility. A participant from a health post commented, “Another thing is since we don’t have a standard referral form, some information may be left out, which is dangerous” (ORO-HP). Data were recorded on patient cards, log books, booklets, referral forms, and plain letter paper. It was unclear how data were tabulated, collected, and maintained. Health posts played a critical role in gathering data and documenting patient visits. At the *kebele* (ward) level, data were recorded in 2 places: the booklet of diagnosis guidelines, and the newborn registration log books (for future follow-up by HEWs for antenatal care). Health post staff were aware that proper documentation informed quality supervision and performance monitoring.

#### Information use

Ongoing performance monitoring appeared to be happening especially in higher-level facilities based on observation, but not necessarily monitoring data. Several respondents supported the idea of performance monitoring and called for more: “Such things should not be periodic, it should be continuous and the top leaders should design a control system for lower-level implementers. So it is good if it has monitoring and evaluation” (AA-HC). They recognized that performance monitoring could reveal training effectiveness. One urban health center spoke about a strong performance monitoring link between the hospital and health center, and with the FMOH.

### Access to essential medicines, supplies, and equipment

#### Equipment functionality and distribution

Problems with dysfunctional equipment and ineffective equipment distribution systems were widespread. Not only was equipment not being purchased, but when purchases were made without consulting or involving providers, they risked being unusable. In the past, nongovernmental organizations provided support to borrow equipment, but this was not a sustainable solution.

Equipment, materials, and medications were unavailable, expired, or broken at all levels of the system. For the purposes of this report, ambulances are classified as equipment because respondents referred to them as such. Few facility leaders said they were well equipped. Problems at facility levels were as follows:

- Health posts: No available weight scale, timer, breath control, blood pressure monitor, radiant heater, phone network, or ambulance, and incomplete medication supplies. Some equipment was provided by nongovernmental organizations but not by the FMOH; some equipment was broken or very old.
- Health centers: No available oxygen supply, radiant heater, incubator, ambulance, phone network, lab reagents, soap, water, or towels; medications were expired or limited. Almost every health center respondent said that ambulances were one or more of the following: unavailable or delayed, lacked oxygen, relied on motorcycles, or experienced big delays; only one health center said ambulances were not a problem.
- Hospitals: No available infusion syringe pump, portable X-ray, oxygen tubes, pulse oximeter, bag valve mask resuscitator, mechanical ventilator, continuous positive airway pressure, incubator, phone, diapers, baby clothes, or personal protective equipment for staff exposed to radiation and infection. Newborns were exposed to infection by sharing beds, incubators, and phototherapy machines. There were shortages of ambulances and NICU supplies, such as infusion syringe pumps, nasal prongs for preterm, bag valve mask resuscitators, vitamin K, and glucometers.

### Health financing

Respondents were nearly unanimous that there was no sustained financial support for preterm, LBW, and sick newborn care in facilities. Hospital leaders talked about being able to include it in the *general* budget, but they asserted that there was no *separate* funding support for programs for preterm, LBW, and sick newborns. One health center representative observed: “I think there needs to be a properly budgeted program which rigorously works to increase awareness in the society. Simultaneously, the health center needs to support facilities and even ambulances so that it can provide the level of health care service promised to the community” (ORO-HC). Funding needs at health centers and hospitals included: specialist salaries, printing of guidelines and policies, basic sick baby supplies (e.g., milk and clothing), transportation for low-income patients (other than transfer ambulances), food for patients and parents, phone service for providers. In the case of health posts, earmarked funds are necessary if there were no actual programs for the care of preterm, LBW, and sick newborns.

### Leadership and governance

#### Guidelines, policies, and protocols

Participants were asked a series of questions about guidelines, clinical standards, protocols, or training modules relevant to prevention, management, or care of preterm, LBW, and sick newborns—specific to the level of facility they supervised. Responses were analyzed to assess how the facility leadership prioritized use of guidelines and to what degree guidelines were available and being used. One big challenge was that leadership across facilities interpreted the concepts of guidelines, policies, and manuals differently. At health posts, references to guidelines primarily included training manuals and chart booklets, while at health centers and hospitals, guidelines might include published manuals, guidelines, and protocols. Availability of guidelines varied widely across facility levels, within the same level but between facilities, and even within facilities. There was no consistency anywhere in the system regarding guidelines for care of preterm, LBW, and sick newborns. Perhaps the most widely available guidelines were related to referrals, some of which were very simplified at the health post level and accessible in chart booklets. This is not to say that other guidelines did not exist, but they were not universally available. Most importantly, if newborn guidelines were available, they were not specific to preterm, LBW, and sick newborns.

Use of guidelines depended primarily upon availability. When guidelines were available, they were seen as an important resource: “We provide the guideline for the assigned provider at the unit and we make sure the guideline stays in the unit; we even have a locker for that so to make sure anybody else assigned at that unit can access the guideline. Because we can’t work without the guideline, the guideline is required critically. The guideline and the log book are inseparable documents” (AMH-HP). Guidelines were used for assessment and referral, resuscitation, parent access to baby, complicated case management, and treatment. One health center representative spoke about posting key points from the guidelines in the medical compound for easy reference. Some spoke about using outdated guidelines because they saw that as better than no guidelines at all. Nearly all those who were aware of, and knowledgeable about, guidelines and policies found them clear and understandable.

For those who did not use or adhere to the guidelines, across all facility levels, reasons ranged from poor dissemination to outdatedness. Respondents recognized the need for ongoing guideline dissemination: “These materials—according to the system, it could be from the ministry of health to the health bureau to the sub-cities and *woredas*—should flow to us timely. And the updates should be properly introduced and training the providers is needed to introduce the providers with new materials so that they can primarily focus on the programs” (AA-HC). When asked about what might facilitate expanded usage, participants had several key suggestions. Challenges and facilitators for guideline use are in Table 5.

**Table 5:** Guidelines for care of preterm, LBW, and sick newborns: challenges and facilitators to provider use

Challenge	Facilitator
Lack of adequate dissemination; often disseminated to individuals rather than facility units; providers removed guidelines from facility for personal use	Fast dissemination; suggest using schools for dissemination; dissemination to facility units; leaflets and flyers as distribution materials
Lack of updated guidelines	Timely updates: publication and via internet
Guidelines did not match well to professional specialty or skill level	Complex cases require guidelines for treatment; Guidelines promotes adherence
Lack of staff knowledge, which may also have manifested as resistance to policies	Performance monitoring and feedback to staff not using guidelines
Lack of supplies, equipment, and infrastructure renders guidelines unusable	Equipment provision for the delivery of care
Lack of periodic professional training	Ongoing training, including in-service
Staff “too busy” to follow manuals; work overload	
Lack of relevance to community needs especially at lower-level health facilities	

#### Supervision and feedback

Staff supervision and feedback were valued by participants, but responses suggested the feedback needs to be systematized and bolstered at all levels across and within facilities. Feedback was primarily through patient referrals. Feedback at catchment meetings was recognized as an opportunity to provide guidance on areas for improvement as well as recognition of accomplishments from the hospital to the health center. Similarly, feedback between health posts and Health Development Army leaders offered opportunities to reduce the number of out-of-facility births. Supportive supervision and mentorship were valued but appeared to come primarily from external sources rather than from direct supervisors or leadership. More stringent supervision and mentoring were needed universally, and especially in NICUs, where high-functioning staff were critical. At the health post level, participants requested that district-level supervisors support health posts when supporting programs from tangential sectors such as agriculture, and also, that they provide follow-up supervision and feedback after trainings.

#### Leadership priorities

Participants were in nearly universal agreement that maternal and child health was a focus priority for the FMOH, citing evidence that emphasis was placed on NICU training, referral, and transport issues. The link between hospitals and health centers helped to maintain the maternal and child health focus. For maternal and child health to be considered a priority focus, however, the FMOH would need to secure additional funding support (including funds to meet newborns’ needs); establish an organized program for preterm, LBW, and sick newborns; and ensure that leadership would drive the program forward. Although respondents noted that treatment and management of preterm, LBW, and sick newborns were considered FMOH priorities, a great gulf remained between stated priorities and availability of materials, recommendations, and training to implement programs. As one respondent stated, “The government has prioritized maternal and child services by recognizing preterm or LBW newborns’ need for early treatment and management, but we are not providing all necessary services here for newborns. .. For the health center to improve service, there should be a protocol; we will get confused to treat or manage such cases in current situations. We will be more confident when necessary materials are adequately available. .. In addition, training should be provided to professionals because knowledge is important for service. Knowledge leads to skill” (AMH-HC).

### Community engagement

Community engagement is not one of the WHO health systems building blocks, but it is vital to providing ongoing care. Participants reported that community engagement and awareness were accomplished through key individuals in the communities: Health Development Army volunteers, religious leaders, traditional birth attendants, and midwives. As one leader suggested, “We should select some influential family members and community members and make them part of trainings to disseminate the information to the community through the community members” (AMH-HP). Additionally, providers recognized that responding to extended families in hospital settings and relying on women who had successfully delivered in facilities were informal ways to spread goodwill between the health system and community. As one health center participant explained, “The most difficult thing for us is that women seem to believe and rely on what they talk about amongst themselves rather than what we teach them. For them we are regarded as outsiders, so we try to make those women from the community who have had health care services speak out on our behalf, and it has proved to be successful” (ORO-HC). Engagement was seen as particularly difficult when the community was mobile. Topics recommended for community awareness included home birth risks, importance of medical care for newborns, and family planning. Participants also acknowledged that leaders must allocate budgets to create awareness.

Some staff at facilities described efforts to engage the community by showing respect for cultural traditions, like coffee ceremonies and porridge making, and by observing Neonatal Day as a holiday. These efforts to draw mothers and families into the health system were up against the strong pull of traditions that limit access to facilities, including prohibiting mothers from leaving home for 40 days following birth, and concerns about babies or mothers dying in a facility far from home.

Communication with families was recognized as vital to health system readiness to serve families of preterm, LBW, and sick newborns. Participants reported that providers’ communication with families in clinical settings varied widely depending on whether responding to their questions or providing updates or information. Communication may have varied widely also by facility type or site. Table 6 shows how respondents saw communication playing out between providers, facilities, and families.

**Table 6:** Communication between families and facility staff: domains and subdomains

Domains	Subdomains
Updating mother/father, sometimes family	Updating done frequently but not during emergency procedures
	Recognition that good updating was part of compassionate and respectful care
	Communicating bad news to parents could be very challenging
	Responsibility for updating parents varied by specialist, general practitioner, or nurse, depending on who was attending to the baby
Parent/family questions policy	Parents were encouraged to ask about newborns anytime; questions from extended family were also answered on demand
	Where policies existed about communicating baby status to parents, they were usually followed; some facilities had no policy
Hospitals and health centers established some formal lines of communication with patients	One medical director interviewed patients directly for performance monitoring
	Midwives were responsible for educating inpatients about postnatal care, newborn care, family planning, etc.

### Programs for preterm, LBW, and sick newborns: existence and components

Participants were asked about the existence and components of programs for preterm, LBW, and sick newborns. Most respondents said their facility had no program of which they were aware. Health post leaders mentioned programs for referral and postnatal education, and health center personnel mentioned equipment (heater, ambulance, and booklets), supervision, the referral process, and previously existing programs. Hospital leaders discussed complex medical programs they currently had. All respondents recognized the importance of focused programs for preterm, LBW, and sick newborns and requested support. As one person said, “First the programs should be initiated; I don’t think the programs are beyond paper” (AMH-HC). Another called for a coordinated effort: “First, there should be trained personnel, then materials that help to provide services for newborns should be adequately available including medications. .. moreover, this program should have a focal person who works on maternal and child health. .. and a strengthened follow-up system must be placed. I think different stakeholders including *woreda*, zone health departments, and partner organizations must be involved to provide regular support” (AMH-HP).

#### FMOH support for preterm, LBW, and sick newborn programs

The FMOH has clearly stated that maternal and child health is a public health priority, but that has not positively affected staff strengthening, funding, or materials provision that would support *initiating* or *continuing* programs for preterm, LBW, and sick newborns. Respondents unanimously agreed that no funds were available from the FMOH for initiating any kind of program for preterm, LBW, and sick newborns at any facilities. Similarly, no respondents were aware of any staff-related support for preterm, LBW, and sick newborn program activities, with the exception of limited training opportunities. Responses were nearly unanimous that there was no support for materials or training guides. A few people referred to initial training or booklets they had received, but they were primarily for midwifery training on newborn care rather than care for preterm, LBW, and sick newborns.

The lack of sustained support for preterm, LBW, and sick newborn programs created a reliance on nongovernmental organizations to fill that gap. The FMOH has not consistently supplied equipment or guides to support ongoing preterm, LBW, and sick newborn programs. Heaters have been supplied to some facilities, but there was also a call for newborn clothing and medications. One person referred to the unsustainable practice of borrowing equipment, and others described equipment that may have gone unused due to lack of training. Table 7 presents key findings and recommendations.

**Table 7:** Key findings and recommendations related to World Health Organization building blocks

WHO Building Block	Key Finding	Recommendation
Service delivery	• Lack of reliable power and water across facility levels • Lack of space for preterm, LBW, and sick newborns and their mothers	• Create separate building/rooms specially designed for preterm, LBW, and sick newborn care, with spaces for mothers/ caretakers • Have backup power source (generator) • Have a water reservoir
Health workforce and human resources	• Shortage of adequate and well-trained health professionals of almost every category at all levels of health facilities • Neonatologists/pediatricians and neonatal nurse specialists in the country were few in number and concentrated in tertiary centers • Trainings and national programs supporting them were integrated with general newborn care and not specifically focused on preterm, LBW, and sick newborn care	• Provide continuous workforce training, motivation, and support to boost skills and commitment in the face of a highly demanding environment of intensive and advance newborn care • Address health care workforce shortages within facilities to meet adequate staffing levels to provide the necessary labor-intensive inpatient care for newborn • Continue to recognize and support health extension workers
Health management information systems and M&E	• M&E data were collected at various levels within the health system inconsistently and irregularly • Available data were not always used for performance monitoring and quality improvement	• Improve the consistency and quality of data collection and use of data at all levels of health system
Access to essential medicines, supplies, equipment	• Shortages of medical supplies, equipment and essential medications were widespread in health facilities at all levels • They were unavailable, broken, or inappropriate for use	• Prioritize procurement and maintenance of critical supplies and equipment
Financing	• Funding allocated for system readiness to care for preterm, LBW, and sick newborns was needed in nearly all facilities at all levels • Specific funding needs included specialist salaries, printing of guidelines and policies, basic sick baby supplies (e.g., milk and clothing), transportation for low-income patients (other than transfer ambulances), food for patients and parents, phone service for providers.	• Fund a program specifically to support preterm, LBW, and sick newborn care at the facility level, which may help to alleviate challenges and ultimately improve the available care
Leadership and governance	• Gap in the availability of guidelines and protocols specifically targeting preterm, LBW, and sick newborns • Staff supervision and feedback were valued by participants, but responses suggested they need to be systematized and bolstered at all levels across and within facilities • Supportive supervision and mentorship were valued but appeared to come primarily from external sources rather than from direct facility leadership	• Develop evidence-based, up-to-date guidelines and protocols specific to the care of preterm, LBW, and sick newborns, and communicate them across the system tier with appropriate supervision • Develop more stringent supervision and mentoring, especially in NICUs where high-functioning staff are critical
Community engagement	• Key facilitators and information disseminators/influencers identified in the study were the Health Development Army, community and religious leaders, and mothers and families who had positive experiences or outcomes of care • Showing respect for the community’s traditions was recognized as an effort to positively change the perception of the community	• Improve awareness through health education, peer modeling, and dissemination of good experiences

*Abbreviations: LBW* low birth weight, *M&E* monitoring and evaluation, *NICU* neonatal intensive care unit, *WHO* World Health Organization

## Discussion

This qualitative study assessed Ethiopia’s health system readiness to support public health facility care of preterm, LBW, and sick newborns. Each of the WHO health system building blocks were considered, and findings revealed that public hospitals, health centers, and health posts faced significant facility and system challenges in all 7 components, making optimum care for preterm, LBW, and sick newborns difficult. Unlike other studies looking at multiple countries, in this study we gathered in-depth information across 3 economically and geographically diverse regions of one country. The findings from this study provide evidence to inform action steps to address complex system-level challenges.

Over the past decade, WHO and others have given much attention to health systems strengthening [7, 15], and the Ethiopian government has reaffirmed commitments and consolidated gains [22]. We relied on facility leadership—the immediate recipients of health system inputs to facilities—as our main data source, building on findings from other studies that health care professionals are key information sources to monitor progress in health systems [23, 24].

### Health service delivery infrastructure and readiness

Ethiopia has achieved significant expansion of all levels of public health facilities in the last 2 decades [17]. Facilities are reported to be well equipped to provide care, but most cited problems in one or more critical areas that inhibited effective provision of care. Reliable power and water have remained major problems, especially in lower-level facilities [25]. Space in health facilities for newborns in need of special care, including space for mothers to stay with their babies, was either absent, inadequate, or lacked privacy. Similar problems were reported in other African and Asian countries [9].

### Workforce/human resources

Health workforce shortages were found to be a critical obstacle to delivering quality care for preterm, LBW, and sick newborns. As found in other reports [8, 9], nationwide shortages of adequate and well-trained health professionals of almost every category were found at all levels of health facilities. The problem was worse in rural and remote regions of the country and at the lowest levels of the health system. The number of neonatologists, pediatricians, and neonatal nurse specialists in the country were few and concentrated in tertiary centers [26]. This problem offered 2 equally important solutions: produce more graduates, and continuously strengthen the capacity of existing staff working in remote and lower-level health facilities. Trainings and the national programs supporting them were integrated with general newborn care and not specifically focused on preterm, LBW, and sick newborn care [27]. Findings also emphasized the important role of HEWs in identifying and referring preterm, LBW, and sick newborns, and the need for recognition and continuous support of HEWs.

### Supplies, equipment, and essential medications

Shortages of medical supplies, equipment, and essential medications were a widespread problem in health facilities at all levels. They were often unavailable, broken, or inappropriate for use. In another report, fewer than half of all facilities had most supplies and equipment needed for preterm, LBW, and sick newborns, and these facilities were primarily specialized hospitals rather than general and primary hospitals [28]. In another report, 35% of Ethiopian health facilities experienced stock-outs of essential medicines, and approximately 8% of medicines in stock had expired [29].

### Health financing

This study showed that specific funding allocated for system readiness to care for preterm, LBW, and sick newborns was needed in nearly all facilities at all levels. For families, the birth of preterm, LBW, and sick newborns could be financially catastrophic. Shifting from a reliance on out-of-pocket payment to prepayment and risk pooling is a critical part of the health financing transition that most countries go through as they get richer. The Ethiopia health care financing strategy aims to replace user fees with funding from the central government. This is expected to boost utilization of services and consequently increase demand on resources [30]. The health care financing strategy targets reduction of out-of-pocket expenditures for direct clinical care services costs, however, and does not include indirect costs for supplies in the form of clothing and formula milk for feeding (when indicated) and emergency transportation costs—which is important for saving lives in cases of emergency by facilitating travel to health facilities.

### Leadership and governance

The availability of guidelines, policies, and protocols, and the practice of supervision and feedback were used as measures of effective leadership and governance. There was a notable gap in the availability of guidelines and protocols specifically targeting preterm, LBW, and sick newborns. From a recent analysis of health system bottlenecks, policy documents in circulation among senior officials were not disseminated to lower-level managers who did not always adhere to guidelines for special newborn care [9]. This study, too, found that in Ethiopia, narrow dissemination and outdated materials contributed to poor adherence to national protocols and guidelines. Another study in Ethiopia reported that hospital activities were poorly supported with a lack of updated or appropriate guidelines and protocols [31].

Staff supervision and feedback were valued by participants, but responses suggested they need to be systematized and bolstered at all levels across and within facilities. Most feedback was related to referrals, and participants spoke positively about effective systems between facilities, especially case-specific feedback. Functional referral systems are crucial for the referral of all patients who need care that cannot be provided in the health facility. An essential component of quality of care for preterm, LBW, and sick newborns is proper handover to the receiving facility and feedback to the sending facility when completing a referral. This is a global standard for improving quality of service for preterm, LBW, and sick newborns [32].

Supportive supervision and mentorship were valued but appeared to come primarily from external sources rather than from direct facility leadership. More stringent supervision and mentoring were suggested universally and especially in NICUs where high-functioning staff are critical. In a study in India, nursing staff felt that there were not enough proficient providers to mentor the nurses to safely perform skills, and they identified lack of mentorship as an obstacle for improving service delivery for preterm, LBW, and sick newborns [33]. Ongoing on-site training, mentoring, and supervision were also cited as absolutely necessary to maintaining the knowledge and motivation of mid-level health care providers and reducing neonatal mortality [34, 35].

Ethiopia’s National Neonatal and Child Survival Strategy aims to reduce the neonatal mortality rate from 28 deaths per 1000 live births to 11 deaths per 1000 live births [6]. Although some interventions like KMC, which were defined in this strategy, may have benefited preterm, LBW, and sick newborns, existing programs were not specifically focused on supporting the care of such newborns in all levels of health facilities.

### Data monitoring and evaluation/health management information systems

This study showed inconsistent and irregular monitoring and evaluation data collection at different levels within the health system. Available data were not always used for performance monitoring and quality improvement. The use of health management information systems is important in developing health care services, but many LMICs struggle with data quality problems resulting in incomplete, inaccurate, and untimely information, which is not useful for health decision making [36]. In Ethiopia where the health management information system is the major source of health information [37], the overall data quality was found to be below international standards [38, 39]. These findings also showed the need for improvements in data quality and usefulness.

### Community engagement

This study revealed that community engagement was considered an important component of serving families with preterm, LBW, and sick newborns. The key facilitators, information disseminators, and influencers identified in the study were the Health Development Army, community and religious leaders, and mothers and families who had had positive experiences or outcomes of care. Showing respect for the community’s traditions was recognized as an effort to positively change the perception of the community. Community engagement in a mobile society was found to be a challenge. The findings were in line with the FMOH strategy to reduce overall neonatal mortality [6]. While engaging community in the care of preterm, LBW, and sick newborns is reported as a major obstacle in many countries [9], a recent study in Malawi showed a shift in perception of community members through health counseling, peer modeling, and personal success with KMC [40].

### Limitations

The input from facility leaders reflects their experiences and expectations, and possibly their optimism or frustration. Expanding the sources of information about the functioning of the health system was beyond our resources. Additional evidence is available from existing hospital data, central reporting systems, and central government representatives. The proportion of providers in facility leadership roles who participated in the interviews may not be representative of all clinicians and providers in the facilities. Information obtained from interviews was not verified by observation.

## Conclusion

The Ethiopian health system has challenges in terms of its readiness to support health facilities to provide quality care for preterm, LBW, and sick newborns. Challenges were reported in all 7 WHO health system building blocks—leadership and governance, health financing, health workforce, essential medical products and technologies, health service delivery, health management information system, and community partnership. Many of the readiness challenges across the health system seem to stem from the inadequate emphasis given to the special care required for preterm, LBW, and sick newborns.

## Recommendations

Based on the findings of this study, we propose the following recommendations and suggested actions (see Table 8).

**Table 8:** Recommendations and actions to improve health system readiness for preterm, LBW, and sick newborns

Health System Building Blocks	Recommendations	Suggested Actions
Service delivery/ infrastructure Supplies/ equipment	• Consideration of critical infrastructure (space, power, water) and equipment to meet the specific needs of preterm, LBW, and sick newborn care	• Create separate building/rooms specially designed for preterm, LBW, and sick newborn care with space for mothers/caretakers • Have backup power source (generator) • Have a water reservoir • Prioritize procurement and maintenance of critical supplies and equipment
Workforce	• Continuous training, motivation, and support for the workforce are necessary to boost skills and commitment in a highly demanding environment of intensive and advance newborn care • Addressing health care workforce shortages within facilities to meet adequate staffing levels for labor-intensive inpatient care for newborns	• Train adequate number of doctors and nurses with neonatal care skills • Conduct refresher training for health workers working in neonatal units • Develop strategies to appropriately remunerate and incentivize neonatal health workers • Provide regular supportive supervision • Recognize the role of health extension workers
Governance/ leadership	• Evidence-based, up-to-date guidelines and protocols specific to the care of preterm, LBW, and sick newborns should be developed and communicated across the system tiers with appropriate supervision	• Develop guidelines and protocols that are specific to preterm, LBW, and sick newborn care and that match skill levels of health workers • Update the guidelines in a timely manner • Disseminate the guidelines to all facilities caring for preterm, LBW, and sick newborns
Health financing	• A program specifically supporting and funding preterm, LBW, and sick newborn care at the facility level may help to alleviate the challenges and ultimately improve the available care	• Develop sustainable source of funding earmarked for preterm, LBW, and sick newborn care at the facility level • Develop mechanisms for incorporating baby food and clothing in medical supplies
Data M&E/ health information system	• Improve the consistency and quality of data collection and use at all levels of health system	• Improve data collection and storage through continuous training and supervision • Monitor data quality through regular feedback and follow-up • Improve data usage at all levels of health system
Community engagement	• Increase community engagement through awareness creation	• Improve awareness through health education, peer modeling, and dissemination of positive experience • Improve family experience at health facilities • Create culture-responsive health care environment

*Abbreviations: LBW* low birth weight, *M&E* monitoring and evaluation

## Supplementary information

**Additional file 1.** Key Informant Interview Guide Facility Leadership.doc, qualitative interview instrument.

## Data Availability

The qualitative data, individual stories and narratives have been collected in personal circumstances. Informants were assured that their contribution will remain confidential to the research project and will not be shared.

## Abbreviations

FMOH: Federal Ministry of Health
HEW: Heath extension worker
HMIS: Health management information system
KMC: Kangaroo mother care
L&D: Labor and delivery
LBW: Low birth weight
LMICs: Low- and middle-income countries
M&E: Monitoring and evaluation
NICU: Neonatal intensive care unit
USAID: United States Agency for International Development (USAID)
WHO: World Health Organization

## References

[1] Chawanpaiboon S, Vogel JP, Moller A, Alkema L (2019). Global, regional, and national estimates of levels of preterm birth in 2014: a systematic review and modelling analysis. Lancet Glob Health.

[2] World Health Organization (2017). Global Health Observatory (GHO) data.

[3] United Nations Children’s Fund (UNICEF), World Health Organization; The World Bank; United Nations, Department of Economic and Social Affairs, Population Division; United Nations Economic Commission for Latin America and the Caribbean Population Division. In: Levels and trends in child mortality: Report 2013. New York, NY: UNICEF; 2013. https://www.who.int/maternal_child_adolescent/documents/levels_trends_child_mortality_2013.pdf?ua=1. Accessed 25 Mar 2019.

[4] Liu L, Oza S, Hogan D (2016). Global, regional, and national causes of child mortality in 2000-15: An updated systematic analysis with implications for the sustainable development Goals. Lancet..

[5] United Nations Interagency Group for Child Mortality Estimation, and UNICEF (2017). Levels & trends in child mortality: report 2017: estimates developed by the UN Inter-Agency Group for Child Mortality Estimation.

[6] Maternal and Child Health Directorate, Federal Ministry of Health (FMOH), Federal Democratic Republic of Ethiopia (2015). National strategy for newborn and child survival in Ethiopia, 2015/16–2019/20.

[7] World Health Organization (WHO) (2010). Monitoring the building blocks of health systems: a handbook of indicators and their measurement strategies.

[8] Dickson KE, Simen-Kapeu A, Kinney MV, Lancet Every Newborn Study Group (2014). Every newborn: health-systems bottlenecks and strategies to accelerate scale-up in countries. Lancet..

[9] Moxon SG, Lawn JE, Dickson KE (2015). Inpatient care of small and sick newborns: a multi-country analysis of health system bottlenecks and potential solutions. BMC Pregnancy Childbirth.

[10] American Academy of Pediatrics Committee on Fetus and Newborn (2012). Levels of neonatal care. Pediatrics..

[11] World Health Organization (WHO); March of Dimes; The Partnership for Maternal, Newborn & Child Health; Save the Children (2012). Born too soon: the global action report on preterm birth.

[12] Allen MC, Cristofalo EA, Kim C (2011). Outcomes of preterm infants: morbidity replaces mortality. Clin Perinatol.

[13] Ellsbury DL, Clark RH, Ursprung R, Handler DL, Dodd ED, Spitzer AR (2016). A multifaceted approach to improving outcomes in the NICU: the Pediatrix 100 000 Babies Campaign. Pediatrics.

[14] O'Brien BC, Harris IB, Beckman TJ, Reed DA, Cook DA (2014). Standards for reporting qualitative research: a synthesis of recommendations. Acad Med.

[15] World Health Organization (WHO) (2007). Everybody's business: strengthening health systems to improve health outcomes: WHO's framework for action.

[16] Population Division, Department of Economic and Social Affairs, United Nations (UN) (2017). World population prospects: the 2017 revision. Key findings and advance tables.

[17] Ministry of Health (MOH), Federal Democratic Republic of Ethiopia (2010). Health Sector Development Programme IV: 2010/11–2014/15.

[18] Morse JM (1995). The significance of saturation. Qual Health Res.

[19] NVivo qualitative data analysis software [computer program] (2018). Version 12; QSR International Pty Ltd.

[20] Central Statistical Agency (CSA), Federal Democratic Republic of Ethiopia; ICF (2016). Ethiopia Demographic and Health Survey 2016. Addis Ababa.

[21] Population and Housing Census of Ethiopia Administrative Report. Addis Ababa, Ethiopia: Central Statistical Authority; 2012. http://unstats.un.org/unsd/censuskb20/Attachment489.aspx?AttachmentType.

[22] Manyazewal T, Oosthuizen MJ, Matlakala MC (2016). Proposing evidence-based strategies to strengthen implementation of healthcare reform in resource-limited settings: a summative analysis. BMJ Open.

[23] Olukoga A, Bachmann M, Harris G, Olukoga T, Oluwadiya K (2010). Analysis of the perception of institutional function for health sector reform in Nigeria. Int Health.

[24] Ganjian S, Dowling PT, Hove J, Moreno G (2015). What physicians from diverse specialties know and support in health care reform. Fam Med.

[25] Ethiopian Public Health Institute (EPHI); Federal Ministry of Health, Federal Democratic Republic of Ethiopia; ICF International (2014). Ethiopia service provision assessment plus survey 2014.

[26] Federal Ministry of Health (FMOH), Federal Democratic Republic of Ethiopia (2016). National human resource for health strategic plan for Ethiopia 2016-2025.

[27] Federal Ministry of Health (FMOH) Federal Democratic Republic of Ethiopia (2018). Basic emergency obstetric and newborn care training manual.

[28] Ethiopian Public Health Institute (EPHI) (2017). Federal Ministry of Health, Federal Democratic Republic of Ethiopia; Averting Maternal Death and Disability, Columbia University Mailman School of Public Health. Ethiopian emergency obstetric and newborn care (EmONC) assessment 2016: final report.

[29] Federal Ministry of Health (FMOH) (2015). Health Sector Transformation Plan.

[30] Federal Ministry of Health (FMOH). National Health Care Financing Strategy, 2015-2035 (2015). Addis Ababa.

[31] Manyazewal T (2017). Using the World Health Organization health system building blocks through survey of healthcare professionals to determine the performance of public healthcare facilities. Arch Public Health.

[32] World Health Organization (WHO) (2016). Standards for improving quality of maternal and newborn care in health facilities.

[33] Campbell-Yeo M, Deorari A, McMillan DD (2014). Educational barriers of nurses caring for sick and at-risk infants in India. Int Nurs Rev.

[34] Neogi SB, Khanna R, Chauhan M (2016). Inpatient care of small and sick newborns in healthcare facilities. J Perinatol.

[35] Brantuo MN, Cristofalo E, Meheš MM (2014). Evidence-based training and mentorship combined with enhanced outcomes surveillance to address the leading causes of neonatal mortality at the district hospital level in Ghana. Tropical Med Int Health.

[36] Hjemås G, Bråthen R, Vikan ST, Haugen JA (2017). Improving quality on health data: recommendations and guidelines. Based on the case of the health management information system in Malawi and DHIS2.

[37] Ethiopian Public Health Institute (EPHI). National technical guidance for maternal and perinatal death surveillance and response. Addis Ababa, Ethiopia: EPHI; 2017. https://www.ephi.gov.et/images/pictures/National-Maternal-and-Perinatal%2D%2DDeath-Surveillance-and-Response-guidance-2017.pdf.

[38] Teklegiorgis K, Tadesse K, Mirutse G, Terefe W (2016). Level of data quality from Health Management Information Systems in a resources limited setting and its associated factors, eastern Ethiopia. S Afr J Inf Manag.

[39] Yarinbab TE, Assefa MK (2018). Utilization of HMIS data and its determinants at health facilities in east Wollega zone, Oromia regional state, Ethiopia: a health facility based cross-sectional study. Res Rev J Med Health Sci.

[40] Lydon M, Longwe M, Likomwa D (2018). Starting the conversation: community perspectives on preterm birth and kangaroo mother care in southern Malawi. J Glob Health.

