# Supplementary material for: Health system readiness to support facilities for care of preterm, low birth weight, and sick newborns in Ethiopia: a qualitative assessment
Source: BMC Health Serv Res. 2019 Nov 21;19:860. doi: 10.1186/s12913-019-4672-2 (PMC6868682; doi:10.1186/s12913-019-4672-2)
Supplement: Supplementary file 1 — Additional file 1. Key Informant Interview Guide Facility Leadership.doc, qualitative interview instrument. [file 12913_2019_4672_MOESM1_ESM.pdf]

Health system readiness to support facilities for care of preterm, low birth weight, and sick newborns in Ethiopia: a qualitative assessment

Key Informant Interview Guide: Head of Health Facility or District Health Office

Italic: Instructions to interviewer. Complete **Interview Information** *section before the start of the interview.*

Interview Information

Name of health facility: ___________________

Zone of health facility: ___________________

Woreda of health facility: _________________ OR Sub-city of health facility: _________________

Level of health facility:

__ Community

__ Health Post

__ Health Center

__ Primary Hospital

__ Secondary Hospital

__ Tertiary Hospital

Date of interview: _____/ ______/ _______

Month / Day / Year

Name of person conducting interview: _________________

Start of Inteview

Introduction

My name is _________________ and I'm an Interviewer working with St. Paul's Hospital Millennium Medical College and the Every Preemie - Scale Study in Ethiopia. As we mentioned previously, this is a research project to understand the care that is provided in health facilities and in the community to small and sick newborns in Ethiopia, and the structures and factors which influence that care. Today we want to talk with you about the type of care that the health facility you manage provides to newborn babies in general and to preterm, low birthweight and sick babies in particular, and to learn about some of the policies, guidelines and resources which influence this care. We are conducting this study at a variety of health facilities in Ethiopia. We are interested in learning about your experience and your perspectives on the structures and factors that affect health services at your workplace. If there are any questions that you do not wish to answer, or you don't have any experience or thoughts on a certain question, you don't have to answer that question. There are no right or wrong answers to the questions we are asking; we just want to know your thoughts.

You were invited to participate in this study because we believed you have experience which may be informative. We will audio-record the conversation, so that we can focus on the discussion and have an

accurate record to review later in more detail. Researchers on our team will listen to this recording and write down what has been said. Then, we will review this information to understand and summarize the thoughts and experiences which you and others have shared. We are going to take notes during our discussion, to help us recall later the important things which you've said.

Do you have any questions?

First, we want to collect some basic information about you. Then we'll begin our discussion.

Interviewee Profile

1. Age: ______(*years*)
2. Gender:

__ Male
__ Female

3. Highest level of education completed (*select 1*):

__ Less than primary
__ Primary
__ Secondary
__ Associate Degree College
__ Bachelor's Degree at University/College
__ Professional/technical
__ Graduate
__ Post-graduate /Doctoral

4. Qualifications: ____________ (*Degrees, credentials)*
5. Current position/title at this facility: __________
6. Time in current position with this facility: ______ (*years)* and ____ (*months)*
7. Time working at this facility: ______ (*years)* and ____ (*months)*
8. Time working in this profession/field: _____*(years)* and ____ (*months)*

Interview Guide

Now we will begin our discussion about your experiences at your facility you supervise. First, we'd like to discuss availability and use of policies and guidelines.

1. A. Are you aware of any FMOH/RHB policies or strategies relevant to prevention, management or care of preterm/low birthweight or sick newborns that are currently used to guide services in the level of facility you supervise? *Probe*: If so, what are the names of these documents? Any others?

   B. *(Ask only if respondent was aware of any policies/strategies in 1A)* How are these policies used in your facility/in the level of facility that you supervise, if at all?

2. A. Are you aware of any guidelines/clinical standards/protocols or training modules relevant to prevention, management or care of preterm/low birthweight or sick newborns for the level of facility you supervise? *Probe*: If so, what are the names of these documents? Any others?

B. *(Ask only if respondent was aware of any guidelines/standards in 2A)* How are these guidelines/clinical standards/protocols/training modules used in your facility/the level of facility you supervise, if at all?

C. In your opinion, what are some of the main things that make it difficult to adhere to the guidelines for care of preterm/low birthweight or sick newborns? *Probe*: Any other important things which hinder adherence to guidelines?

D. In your opinion, what are some of the main things which have supported or facilitated the adherence to guidelines for care of preterm/LBW/sick newborns? *Probe*: Any other important things which facilitate adherence to guidelines?

3. Are there content areas where you feel it is unclear what the policy or guideline is for the care or services provided to preterm, low birthweight or sick babies or where policies or guidelines are lacking? By "content area" we mean specific services, interventions, treatment modes, medication/drugs, devices, protocols etc.

Probe: Any other content areas where policies or guidelines are lacking?

4. A. Can you explain the process through which the government disseminates new policy or guidelines for care and services for preterm/low birthweight/sick newborns in your facility, including how the change is communicated and how your institution is expected to institute the change?

   B. Does the facility receive any resources or support for instituting new policies or guidelines for preterm, low birthweight or sick newborns?

   C. What types of support from the government, if any, would you say might help in instituting new policies, guidelines or programs successfully in future?

   D. What are some of the key challenges to the dissemination and implementation process?

   E. How would you recommend the process for dissemination and implementation be improved?

5. A. Does the facility have a specific program for preterm/low birthweight newborns? If so, can you explain to me what the program consists of?

   B. Did the facility receive any support or resources from the Ministry to support <u>the initiation</u> of the program for preterm/low birthweight newborns? By "support" we mean things like specialty training opportunities for staff, provision of additional staffing/personnel, receipt of facilitations materials or training guides etc.

Probe: Any other support or resources?

C. Are there funds made available to your facility from the Ministry that target <u>initiation of a new service or program</u> for preterm, low birthweight and sick newborns?

D. What support, resources, or financing, if any, do you think might have been helpful to successfully <u>initiate</u> a <u>new service or program</u> for preterm/low birthweight or sick newborns?

E. Does the facility receive any ongoing support, resources or financing from the Ministry to support ongoing implementation or improvement of services or programs for preterm/low birthweight or sick newborns?

F. What support, resources, or financing, if any, do you think might have been helpful to support ongoing implementation or improvement of services or programs for preterm/low birthweight or sick newborns?

Now we have some questions about the basic infrastructure and set-up at the facility/level of facility you supervise.

6. A. Do you believe there is adequate basic infrastructure available at the facility/level of facility you supervise to implement services for preterm, low birth weight, and sick newborns? By "basic infrastructure" we mean continued un-interrupted power, running water at care locations, and physical space.

   B. Is there continued un-interrupted power available at locations providing care for preterm, low birth weight and sick newborns?

   C. Is there running water at locations providing care for preterm, low birth weight and sick newborns?

   D. Is there adequate physical space at locations providing care for preterm, low birth weight and sick newborns?

We would like to ask you about how prepared the facility is to provide care in various locations with your facility.

7. A. Do you think the facility is prepared to provide routine care for newborns at the time of birth in the labor and delivery ward?

   B. In your opinion, what are the main factors that make it difficult to deliver routine care at the time of birth for newborn babies in the labor and delivery ward? *Probe:* Any other factors?

8. A. For babies who remain in the hospital for concerns or problems with the mother's health, do you believe the facility is prepared to provide routine care for the newborns?

   B. In your opinion, what are the main factors that make it difficult to deliver routine care to newborns who remain in facility due to health problems of the mother? *Probe:* Any other factors?

9. A. For newborns who need special care because they are small or sick, in a neonatal intensive care unit (NICU), special newborn unit (NBU), inpatient ward, or KMC, do you think the facility is prepared to provide safe and effective services?

   B. In your opinion, what are the main factors that make it difficult to deliver safe and effective services for small and sick newborns in the NICU, NBU, inpatient ward, or KMC at this facility? *Probe:* Any other factors?

Now I have some questions about the types of access that parents/family members have to newborns in the facility.

Only ask the questions below that are relevant based on the level of facility the interviewee supervises, and the types of wards/units which are present in the level of facility the interviewee supervises.

Ask only if the facility has a neonatal intensive care unit/NICU

10. A. In the facility you supervise, is there a policy in place that specifies the type and amount of access that a family member/parent can have to a small or sick baby who is receiving advanced care in a NICU? If so, can you describe the policy? For example: parents have 24/7 access to their newborns.

    B. Is there onsite accommodations, or a room where parents can stay the night at the facility if their small or sick newborn is receiving advanced care in a NICU?

Ask only if the facility has an inpatient newborn unit/special newborn care unit

11. A. In the facility you supervise, is there a policy in place that specifies the type and amount of access that a family member/parent can have to baby who is receiving care in a newborn unit? If so, can you describe the policy? For example: parents have 24/7 access to their newborns.

    B. Is there a policy in place to allow in-rooming for the parents (the ability for parents to stay in the room with the baby overnight, or a room for parents to stay the night at the facility) of newborns who are receiving care in a newborn unit?

Ask only if the facility has an kangaroo care unit

12. A. In the facility you supervise, is there a policy in place that specifies the type and amount of access that a family member/parent can have to baby who is receiving care in a newborn unit? If so, can you describe the policy? For example: parents have 24/7 access to their newborns.

    B. Is there a policy in place to allow in-rooming for the parents (the ability for parents to stay in the room with the baby overnight, or a room for parents to stay the night at the facility) of newborns who are receiving care in a newborn unit?

Ask only if the facility has a postnatal ward

13. A. In the facility you supervise, is there a policy in place that specifies the type and amount of access that a family member/parent can have to baby who is receiving care in a postnatal ward? If so, can you describe the policy? Are newborns separated from their family member/parent?
    B. Is there a policy in place to allow in-rooming for the parents (the ability for parents to stay in the room with the baby overnight, or a room for parents to stay the night at the facility) of newborns who are receiving care in a postnatal ward?

Now we'd like to discuss the type of communication that occurs between staff at the facilities and family members/parents of newborns.

14. A. Is there a policy or procedure in place at the facility you supervise about communication to family members/parents about the current status of a newborn who is receiving care at the facility? This might be a policy that states how often a parent/family member is meant to be updated about the status of newborn, whose responsibility it is to update the family member/parent, when updates/communication is meant to occur, the type of information that should be shared with a parent/family member about the newborn's status or treatments etc.

    B. Is there a policy or procedure in place that describes a method for families to ask questions about the care newborns are receiving and the newborn's status? This could be a policy or

procedures that states specific times parents should be encouraged to ask question, specifies who is available and when they are available to answer parents' questions etc.

I have a couple of questions now about how the referral and transfer systems work at the facility you supervise.

15. When there are newborns who are preterm, low birth weight or sick who can't be managed effectively at this facility, what is the process for referral or transfer to the next level of care?

    If health post, ask about referral from health post to health center.

    If health center, ask about referral from health center to primary hospital. If primary hospital, ask about referral from primary hospital to secondary hospital.

    If secondary hospital, ask about referral from secondary to tertiary hospital. If tertiary hospital, ask if there is anywhere the babies might get transferred for specialty care.

    A. Is there a specific policy or procedure in place that states to what facility and what unit in a facility a baby is referred?

    B. What is the criteria for referral?

    C. Is there a standard referral form which gets completed here at this facility?

    D. Describe the method through which medical history or diagnosis and treatment history of the newborn is communicated to the new facility/new providers.

    E. What is the established method for feedback from the transfer facility about the outcome or status of the newborn?

16. Now I'd like to hear about the process for referral or transfer of preterm, low birth weight or sick newborns to this facility from a lower level of care.

    If health post, ask about referral from community to health post.

    If health center, ask about referral from health post to health center.

    If district hospital, ask about referral from health center to primary hospital.

    If secondary zonal hospital, ask about referral from primary district hospital.

    If tertiary hospital, ask about referral from primary district or secondary zonal hospital.

    A. Is there is a specific policy or procedure in place that states from what facilities or units in a facility a baby will be accepted?

    B. What is the criteria for acceptance of the referral?

    C. Is there is a standard referral form which the facility receives upon arrival of the newborn or a standard form which the facility completes when they accept a referral/transfer?

    D. Describe the method through which medical history or diagnosis and treatment history of the newborn is communicated to your facility/providers.

    E. What is the established method to provide feedback about the outcome or status of the newborn to the facility from which they were referred or transferred?

17. A. Thinking about both babies who are referred/transferred here or referred/transferred to

another facility, what are some of things that make this referral/transfer process successful?

B. What are some of the limitations or problems with the current referral/ transfer system? *Probe*: What factors do you think might strengthen or improve the referral system?

Now we have some questions about initiatives or programs designed to improve the quality of care that may have occurred at the facility you supervise.

18. A. Have there been any quality of care (QC/QI) processes initiated for the care of preterm/low birth weight or sick newborns in this facility in the last year? If so, can you describe the initiate?

    Ask only if respondent said there was an initiative
    B. Who initiated this program and who were the partners involved (FMOH, NGO partner, internal facility program etc.)?

    Ask only if respondent said there was an initiative
    C. In your opinion, do you think this quality of care project effectively improved the quality of care provided to small or sick newborns at the facility in a meaningful way?

    Ask only if respondent said there was an initiative
    D. In your opinion, was that QI/QC program sustainable? That is, was the facility able to continue this program in an ongoing fashion?

    Ask only if respondent said there was an initiative
    E. What do you think would help strengthen the sustainability and/or effectiveness of a quality of care (QI/QC) program for preterm and/or sick newborns in your facility?

    F. In your opinion, would the facility benefit from a quality of care (QI/QC) initiative to improve the quality of care for small and/or for sick newborns?

For our last few questions, we are interested in hearing your opinions and perspectives on what is needed in the health system in Ethiopia to improve care for preterm, low birthweight and sick newborns.

19. There are many factors which could be addressed to strengthen the quality of care that is provided to preterm, low birthweight or sick newborns in a facility. I'm going to mention a few potential areas that could be focused on, and I'm interested in your opinion about what you think the most important areas to focus on are in your set-up, to improve the quality of care that is provided to preterm/small/sick babies in this facility. I'm going to show you a list of some potential areas that could be addressed, and I'd like you to tell me what you think the most important of these areas are in your set-up. Then, I'd like to hear some more details about what you chose.

    (Show respondent a printed list of the items below and ask them to choose what they believe are the THREE most important in their setting. Then, ask them to discuss/expand on why they chose what they did).

    - Availability of guidelines and policies to guide what care and services are provided
    - Awareness among the staff about appropriate care
    - Technical competency and training of staff
    - Number and availability of trained staff
    - Leadership and supervision of staff

- Designated space /room/infrastructure
- Availability of basic supplies
- Availability of equipment, machines, technologies
- Awareness among women/families of newborns about the availability and benefit of services
- Barriers to seeking care or accepting care for small babies from women/families

Probe: Are there any other important elements which in your opinion which could be addressed to improve the services for preterm, low birth weight or sick newborns in this facility which we didn't mention?

20. Do you have anything else you'd like to share with us about what influences the care and services the facility you supervise/level of care you supervise is able to provide to preterm, low birth weight or sick newborns?

Thank you for your time and for sharing your valuable experience with us.
